# Molecular Features of the Mesenchymal and Osteoblastic Cells in Multiple Myeloma

**DOI:** 10.3390/ijms232415448

**Published:** 2022-12-07

**Authors:** Nicolas Thomas Iannozzi, Valentina Marchica, Denise Toscani, Jessica Burroughs Garcìa, Nicola Giuliani, Paola Storti

**Affiliations:** 1 Department of Medicine and Surgery, University of Parma, 43126 Parma, Italy; 2 Hematology, Azienda Ospedaliero-Universitaria di Parma, 43126 Parma, Italy

**Keywords:** multiple myeloma, mesenchymal cells, osteoblasts, molecular pathways

## Abstract

Multiple myeloma (MM) is a monoclonal gammopathy characterized by biological heterogeneity and unregulated proliferation of plasma cells (PCs) in bone marrow (BM). MM is a multistep process based on genomic instability, epigenetic dysregulation and a tight cross-talk with the BM microenvironment that plays a pivotal role supporting the proliferation, survival, drug-resistance and homing of PCs. The BM microenvironment consists of a hematopoietic and a non-hematopoietic compartment, which cooperate to create a tumor environment. Among the non-hematopoietic component, mesenchymal stromal cells (MSCs) and osteoblasts (OBs) appear transcriptionally and functionally different in MM patients compared to healthy donors (HDs) and to patients with pre-malignant monoclonal gammopathies. Alterations of both MSCs and OBs underly the osteolytic lesions that characterize myeloma-associated bone disease. In this review, we will discuss the different characteristics of MSCs and OBs in MM patients, analyzing the transcriptome, the deregulated molecular pathways and the role performed by miRNAs and exosome in the pathophysiology of MM.

## 1. Introduction

Multiple myeloma (MM) is an incurable malignant disease marked by the growth of malignant plasma cells (PCs) in the bone marrow (BM) [1]. MM is about 10% of hematologic malignancies and, after lymphomas, represents the second most frequent hematologic malignancy [2]. MM can be preceded by pre-malignant stages named monoclonal gammopathy of undetermined significance (MGUS) and smoldering myeloma (SMM) [3].

Malignant PCs are characterized by various chromosomal alterations, such as hyperdiploidy or non-hyperdiploidy involving translocations (t). Hyperdiploid patients are characterized by trisomies involving odd chromosomes [4]. The non-hyperdiploid group involves patients with primary translocation in the 14q32 region, which contains genes coding for immunoglobulin (Ig) heavy chains (IgH locus) [5]. Primary IgH translocations are found in 40% of MM patients [6].

The most frequent secondary alteration is the amplification (amp) of chromosome region 1q (amp(1q)), found in 40% of MM patients. Particularly, when there are three copies of this chromosome, it is referred to as a gain, and if there are four or more copies, it is reported as an amp [7]. Some of the genes involved in the 1q21 chromosome region are B cell lymphoma (*BCL-9*), interleukin (IL)-6 receptor (*IL6R*), interleukin-2 enhancer binding factor 2 (*ILF2*), myeloid cell leukemia 1 (*MCL-1*) and cyclin-dependent kinase regulatory subunit 1B (*CKS1B*) [8].

Another gene that is significantly important in ordinary cell physiology is the tumor protein p53 (*TP53*). In MM, it undergoes inactivation because it is located on the p arm of chromosome 17, which is often affected by a deletion (del(17p)) [7].

Disease progression is also influenced by deregulation of signaling pathways, such as the *RAS/RAF/MEK/ERK*- (also known as *MAPK*-) pathway, nuclear factor-kB (*NF-kB*-) pathways and the phosphatidylinositol 3-kinase (*PI3K*)/protein kinase B (*AKT*) [9].

The expansion of the MM PCs is supported, in parallel with cytogenetic alterations, by a strong contribution from the BM microenvironment. In fact, MM PCs reside in the BM and their proliferation is fully dependent on their interaction with almost all components of the BM microenvironment [10].

## 2. The Bone Microenvironment: Mesenchymal Stromal Cells and Osteoblasts

The BM microenvironment consists of a cellular and a non-cellular compartment, each of which has components that play distinct but interacting roles in the progression of MM. The cellular compartment includes hematopoietic and non-hematopoietic cells. The first comprises myeloid cells, T and B lymphocytes, natural killer (NK) cells, macrophages and osteoclasts (OCLs), whereas the non-hematopoietic cell types include BM stromal cells, BM-derived mesenchymal stromal cells (MSCs), osteoblasts (OBs), osteocytes, fibroblasts, adipocytes and endothelial cells. The second major area, as represented by the non-cellular component, includes the extracellular matrix and the growth factors, chemokines, different cytokines and exosomes derived from the cellular compartment [11]. Release of chemokines, cytokines and adhesion molecules induces growth and development of cancer cells [12].

Among the non-hematopoietic cell compartment, we focus of the molecular features of MSCs and OBs and their involvement in the pathophysiology of MM.

### 2.1. Mesenchymal Stromal Cells

The term ‘Mesenchymal Stromal Cells’ was introduced by Caplan around 1991 [13], referring to BM progenitor cells. These cells exhibit stemness activity; in fact, they are described as multipotent cells [13]. They represent a group of cells with an appearance very similar to fibroblasts, capable of forming fibroblastic colonies in vitro derived from a single cell and thus referred to as fibroblast colony-forming units (CFU-Fs) [14], with replicative and differentiating potential. Therefore, MSCs exhibit two basic properties: self-renewal and the ability to originate different cell lines. The first characteristic determines the ability to generate a daughter cell with the same stemness characteristics as the parent cell, the second feature allows MSCs to differentiate and generate adipose, cartilage and bone tissue [15].

In addition to the two functional characteristics previously discussed, MSCs must also satisfy phenotypic criteria, including positive expression of the markers CD105, CD90 and CD73, and non-expression of CD11b, CD14, CD19, CD34, CD45, or CD79α and HLA-DR surface molecules [16,17].

As an immunophenotype, MSCs also express transmembrane glycoproteins, namely tool-like receptors (TLRs), which are responsible for detecting foreign pathogens and endogenous damage signals [18]. TLRs can be present either on the cell membrane (TLR1, TLR2, TLR4, TLR5, TLR6 and TLR10) or in the intracellular part, such as endoplasmic reticulum and endosomes (TLR3, TLR7, TLR8 and TLR9) [19].

Moreover, BM-MSCs have the ability to differentiate into OBs, osteocytes, adipocytes, chondrocytes, muscle cells and reticular fibroblasts [20]. New RNA-seq studies have suggested that MSCs express the genetic profiles needed for osteoblastic and adipocytic differentiation but show more pronounced transcriptome change during differentiation into adipocytes [21].

Physiologically, these specialized cells are highly important since they ensure the appropriate BM organization and regulate the formation and resorption of bone [22].

### 2.2. Osteoblasts

OBs are derived from MSCs, with a cubic shape, and are the ones responsible for bone formation.

OBs are cells that cover bone and store the mineralized matrix of the skeleton through a sequence of steps. In fact, from MSCs, OBs progenitors specifically express the genes enumerated above and proceed into a proliferative phase. At this stage they are considered pre-OBs and there is an increase in alkaline phosphatase (*ALP*) activity [23]. Later, there is an increase in osteocalcin (*OCN*), bone sialoprotein (*BSP*) and collagen I (*COL1*) which marks the terminal transition from pre-OBs to mature OBs [24].

During the bone formation process, OBs also operate by depositing a dense organic extracellular matrix principally formed by *COL1*. This dense matrix will undergo a hardening process with production of hydroxyapatite, an inorganic mineral made of phosphate and calcium [25].

For the differentiation from MSCs, the synthesis of wingless/int1 pathway (Wnt) family members and bone morphogenetic proteins (Bmp) is important. To be considered as a prerogative is also the expression of specific genes, such as osterix (*OSX*), distal-less homeobox 5 (*DLX5*) and runt-related transcription factor 2 (*RUNX2*, also called *CBFA1* or *AML3*) [24].

Below, we describe the significant role of Wnt and Runx2 pathways in osteoblastic differentiation.

#### Osteogenic Differentiation of MSCs: Role of Wnt and Runx2 Signaling

Several studies have demonstrated that Wnt signaling plays a crucial role in the regulation of osteoblastogenesis [26,27]. To date, two pathways (canonical and non-canonical) and nineteen forms of Wnt have been found in humans [28,29].

The canonical Wnt signaling pathway is mediated by β-catenin. This is activated by Wnt 1/3a through linkage to its receptors, frizzled (Fzd) and low-density lipoprotein receptor-related protein (LRP 5/6). Linking stimulates phosphorylation of the glycogen synthase kinase (GSK3)/axin complex; in fact, axin gets moving with the intracellular protein dishevelled (Dsh) to the membrane where it interacts with LRP5/6, and this occurrence leads to stabilization and translocation into the nucleus of β-catenin in a dephosphorylated state. This event results in the activation of the lymphoid enhancer factor (Lef 1)/T cell factor (Tcf) transcription system [30]. The downstream effects of this signaling involve transcription of bone-forming genes, regulating pre-OB differentiation via Runx2 and/or Osx induction [24].

In the absence of Wnt ligand binding, β-catenin undergoes proteosomal degradation; in fact, adenomatous polyposis coli (APC) tumor suppressor protein, scaffold protein axin and GSK-3β form a complex that sequester β-catenin. In this way, it is targeted for polyubiquitination by β-transducin repeat-containing protein (β-TrCP) and its subsequent destruction by the proteosome [30].

The non-canonical pathway is β-catenin-independent; in fact, there is no intracellular accumulation of β-catenin. The non-canonical pathway therefore includes the Wnt/JUN N-terminal kinase (JNK)/planar cell polarity (PCP) and Wnt/calcium pathways [31].

The Wnt/JNK/PCP is triggered by the binding of Wnt5a ligand to FZD family receptors and takes advantage of the orphan receptor tyrosine kinase (Ror) as a co-receptor [32]. GSK-3β phosphorylates Ror1 and Ror2 form a complex with the intracellular DSH to constitute the activated Fzd/Ror complex. This prompts the activation of small GTPases such as Ras homolog gene family member A (RhoA) and cell division control protein 42 (Cdc42). Moreover, Dvl activates Ras-related C3 botulinum toxin substrate (Rac) and lastly JNK [33].

The second non-canonical pathway is the Wnt/calcium pathway [34]. For this second non-canonical molecular pathway, binding of Wnt5a to the Fzd/Ror complex is always necessary. The link activates Dsh and trimeric G-proteins (Gα, β, γ). Afterwards, there is phospholipase C (PLC) activation, which generates inositol 1,4,5-triphosphate (IP3) and diacylglycerol (DAG). IP3 induces Ca^2+^ release, which in turn triggers protein kinase C (PKC), additionally stimulated by DAG, thus initiating nuclear translocation of activated T cell nuclear factor (NFAT) and NF-kB [34].

Wnt signaling is thus a master regulator of bone homeostasis, driving the MSCs differentiation toward the osteoblastic lineage, and it also enhances the survival of these cells [35].

*RUNX2* is the most important transcription factor involved in the process of osteoblastic differentiation and exists as *RUNX1*, *RUNX2* and *RUNX3* [36]. The human OB differentiation is mainly associated with increased Runx2 activity. On the other hand, in mature OBs Runx2 is weakly expressed, demonstrating the importance of early differentiation [37].

Runx2 expression and activity is positively regulated by transcription factors such as Taz, Hoxa10 and Bapx-1, and negatively regulated by Hey-1, Hoxa2, Stat1 and Sox9 [32]. Foxhead box class O family member (FoxOs) proteins are also involved in the OBs differentiation process. FoxO1 regulates the lineage differentiation of OBs. In fact, its overexpression can significantly increase the expression of osteogenic genes such as *Runx2*, *Alp* and *Ocn* in mouse mesenchymal stem cells and MC3T3E1 pre-OBs cells [38,39]. FoxO1 physically interacts with activating transcription factor 4 (ATF4), which is a crucial regulator in bone formation by controlling amino acid uptake and protein synthesis. Indeed, mice with *FoxO1* inactivation showed reduced bone matrix protein synthesis with low OB numbers and subsequent reduced bone mass [40].

An additional factor that regulates osteoblastic differentiation is Osx, also known as SP7. Mice with *Osx* deletion do not survive due to the general absence of bone formation and massive rib cage malformation [41]. Furthermore, Runx2 expression is noted in Osx-deficient OBs, but Osx is not expressed in Runx2-deficient OBs, suggesting that Runx2 regulates Osx [42]. In addition, transcriptional regulation of Osx by NFATc1 has also been demonstrated, thereby regulating bone development [43].

Bmps also regulate Osx expression; in fact, Bmp2 promotes the *Runx2* and *Osx* expression in murine osteoprogenitor and osteoblastic cells [32].

Finally, Runx2 activation upregulates expression of OBs-related genes such as *COL1A1*, *ALP* and *BSP* [44].

## 3. Bone Marrow Microenvironment in Multiple Myeloma

The BM microenvironment represents a highly dynamic niche that can support both malignant transformation and disease progression [45]. It is able to generate a highly inflammatory environment through cytokine release and ensures very intense cellular communication [45]. It also guarantees a high rate of infiltration and migration of MM cells. Therefore, MM depends on the permissiveness of the BM microenvironment, and in this view, there is a bidirectional interaction between neoplastic PCs and the microenvironment. For example, it is now well known that MM patients show aberrant expression of hypoxia-inducible factors (*HIF1α*, *HIF2*) or vascular-endothelial growth factor (VEGF) in relation to the hypoxic status observed in the BM microenvironment [46].

Immune cells such as Th17 lymphocytes, a subset of CD4 T cells, are capable of inducing immunosuppression and are highly present in the BM of MM patients [47]. The recruitment of Th17 lymphocytes into the microenvironment of MM patients is promoted by the upregulation of the chemokine CC-chemokine ligand 20 (*CCL20*) [48]. Furthermore, they are involved in the release of IL-10 and IL-17. IL-17 is a pro-osteoclastogenic cytokine, which induces the rank-ligand (Rankl) production; thus, it contributes to myeloma-associated bone disease [49]. In addition to this cell type, tumor associated macrophages (TAMs) are also found, which are competent enough to produce significant amounts of cytokines [50].

In recent years, a novel heterogeneous population of myeloid suppressor cells (MDSCs) has emerged, which include immature, neutrophilic cells and have a strong immunosuppressive signature. In fact, these cells produce the enzyme arginase that depletes the environment of arginine, an amino acid essential for T lymphocyte activity. In addition, they ensure the expansion of induced Treg [51,52].

MDSCs have also the ability to differentiate into TAMs and into OCLs. OCLs are numerically predominant over OBs in MM, and this underlies the characteristic bone disease observed in MM patients [53]. In fact, in MM, the whole BM organization fails. Indeed, there is a disequilibrium between OBs and OCLs activity.

This evidence, taken together, identifies the BM microenvironment as responsible for proliferation, survival, drug resistance, disease progression and de-coupling of the bone remodeling process [54,55].

## 4. Distinctive Features of MSCs in Multiple Myeloma

MSCs are significantly involved in MM pathogenesis of disease progression, drug resistance, migration of myeloma cells and low osteogenic activity. Several investigators have directed their research into the study of the BM microenvironment, with the aim of identifying important differences between healthy and MM patients.

Several studies have been conducted to try to better understand the functional, molecular and genetic characteristics of MSCs in MM patients. In fact, it has been shown that the MSCs of MM patients (MM-MSCs) have different gene profiles than MSCs from healthy donors (HD-MSCs) [56]. Other groups have proved that the presence of MM cells is able to induce modifications in the phenotype of MSCs, for example, by co-culturing MM cells with MSCs from HDs, they acquire features similar to MSCs obtained from BM of MM patients [57]. In this report, we will examine the role of cytokines, gene expression of HD-MSCs vs. MM patients and the influence of miRNAs on these cells.

### 4.1. Cytokines Profile and Effect of Cytokines on MSCs

Regarding cytokine release, there are striking differences observed between HD-MSCs and MM-MSCs [58]. In fact, there is a larger cytokine production and release in MM-MSCs compared to HD-MSCs, including various interleukins such as IL-6, IL-1b, IL-19 or Vegf [58,59].

IL-6 is the major growth and survival factor of MM cells. Arnulf et al. measured IL-6 levels in the supernatants of MSCs derived from MM patients and HDs, demonstrating a considerable increase in MM patients [58]. The interworking between MSCs and MM cells upregulates the transcription and IL-6 synthesis in MSCs [60]; in turn, IL-6 triggers the proliferation of MM cells following a paracrine loop which is stimulated by the clearance of other molecules and chemokines, including CD40, tumor necrosis factor (Tnf)-a, Vegf, IL-1β and transforming growth factor (Tgf)-β. IL-6 and IL-1β work together in disease progression, because IL-1β, produced in high amounts by MM cells, induces IL-6 production by MSCs [61]. In detail, IL-1β regulates the transcription of IL-6, which is mediated by upregulation of NF-kB activation. IL-6 stimulates the proliferation-inducing ligand (*APRIL*) and B-cell activating factor (BAFF) production, activating *NF-kB/PI3K/AKT* and *MAPK* pathways and thus promoting MM cell survival [62]. TNF-α also induces IL-6 secretion in BMSCs activating NF-kB [62]. Lust et al. demonstrated both in vivo and in vitro that blockade of IL-1β results in decreased IL-6 activity [63,64].

To accomplish its function, IL-6 necessitates binding to its receptor (IL-6Ra) composed of an essential subunit for linkage (gp80) and a portion with the function of signal transducer (gp130). When binding occurs, it causes the activation of several pathways. The *JAK/STAT3/MCL1* (janus kinase/signal transducers and activators of myeloid leukemia cell differentiation transcription/protein) promotes survival and through the *RAS/MEK/MAPK* (mitogen-extracellular signal-regulated kinase/mitogen activated protein kinases) it stimulates cell proliferation. It is also able to activate *PI3K*/*AKT* signaling [65].

Moreover, the cross-talk with the MM cells augments the secretion of activin A in MSCs and also in OCLs. This over-expression is led by an adhesion-mediated JNK activation, reported by a JNK phosphorylation in MSCs. Activin A also inhibits MSC differentiation by a SMAD2-dependent DLX5 downregulation [66,67]. An additional factor with a critical role that is especially expressed in MM patients is Vegf. It is overexpressed by NF-kB, which can be activated by the PI3K/AKT signaling pathway [68]. This angiogenic cytokine is responsible for the formation of neo-vessels from those previously formed [69]. In MM, Vegf is produced by both cancer cells and by MSCs. It has two functions, namely, it stimulates IL-6 transcription by MSCs and promotes migration of MM cells via *MEK/ERK* signaling [70]. In turn, *VEGF* is governed by HIF-1, a pivotal angiogenesis driver, because it induces the mRNA expression of *VEGF* [71].

Moreover, several cytokines and chemokines present in the MM microenvironment influence MSCs. Hepatocyte growth factor (Hgf) is produced by MM cells and promotes proliferation of MSCs, keeping cells undifferentiated [67]. MM cells also produce IL-7 that diminishes Runx2 transcriptional activity and induces the growth factor independent 1 transcriptional repressor (Gfi1) in MSCs that represses Runx2 [72,73]. Similarly, TNF-α has been shown to be active in MM MSCs; in particular, TNF-α is produced by MM cells and induces Gfi-1 expression in MSCs reducing the expression of Runx2 [74].

Recently, it has been reported that macrophage inflammatory protein-1alpha (MIP-1alpha), a CC chemokine produced in large amount of MM patients, increases the level of Rankl expression in MSCs through MAPK and PI3K/Akt pathways [75]. Rankl is also increased in MSCs and OBs by Hgf, which is elevated in MM patients. Tsubaki et al. [76] found that HGF and MM cell supernatants induced Rankl expression in ST2 cells, MC3T3-E1 cells and mouse MSCs; in addition, Hgf increased phosphorylation of Met and NF-κB in the same cells. These data suggest that Hgf promotes Rankl expression in OBs and MSCs via the Met/NF-kB signaling pathway [76].

Lastly, a recent study conducted on adipocyte-derived MSCs (ASCs) showed that seven cytokines (ANG1, ENA-78, EGF, PDGF-AA/AB/BB and TARC) were increased in the plasma of MM patients and separately inhibited the osteoblastic differentiation of HD-ASCs [77].

### 4.2. Gene Expression Signature of MSCs in Multiple Myeloma

Different teams of investigators have tried to identify more highly expressed genes in MSCs from BM of MM patients.

Giuliani et al. examined the genomic profiles and the presence of telomere maintenance mechanism of MSCs. Their results suggest that MSCs do not exhibit malignancy features; thus, they are not part of the tumor clone [78].

In another study, however, by array-based comparative genomic hybridization (array-CGH), genomic imbalances were discovered in regions that contained coding genes. It emerged as deregulated expression for five encoding genes. Among the gene losses were potassium channel tetramerization domain containing 8 (*KCTD8*) and bone morphogenetic protein 10 (*BMP10*), while in the genomic gain in array-CGH there appeared to be increased RNA levels for fibulin 5 EGF-like protein (*FBLN5*), zinc finger protein 217 (*ZNF217*) and inositol-tetrakisphosphate 1-kinase (*ITPK1*) [56]. *FBLN5* may promote the growth of MM cells, while Bmp10 in MSCs may affect their capacity for osteoblastic differentiation [79].

In one of the earliest studies carried out on MSCs, Corre et al. identified 127 distinct genes engaged in the cancer-microenvironment cross-talk. In fact, these genes were expressed differentially in HDs and MM patients. In addition, their ability to lineage osteoblastic origination appeared compromised [59]. The genes identified codified for receptor signaling molecules, for proteins implicated in cell communication, in metabolism, in the cell cycle and in the control of apoptosis. The identified genes comprised amphiregulin (*AREG*), *IL-1β*, dickkopf-1 (*DKK1*), insulin-like growth factor 1 (*IGF1*) and stromal cell-derived factor-1 (*SDF1*) [59]. Moreover, the growth and differentiation factor-15 (*GDF15*) was also more highly expressed in the MSCs of MM patients than in HD-MSCs [59].

Todoerti et al. investigated the transcriptional profiles of MSCs and OBs and their association with the presence of osteolysis in MM patients. They identified dissimilar gene expression profiles in MSCs and OBs of MM patients compared with HD patients [80]. In particular, there was a different gene expression of genes involved in the proliferation and differentiation of MSC cells, which are *HOXB2, HOXB6* and *HOXB7* genes. On the other hand, regarding the gene expression of MSCs and OBs in MM patients with osteolytic lesions compared to the non-osteolytic ones, only MSCs have distinct gene signatures [80].

Moreover, in a more recent study by Schinke et al., it was confirmed that MSC expression diverged in HDs and patients with MGUS, SMM, MM and complete remission (CR) samples. This was possible using a 34-gene model. Of these 34 differentially expressed genes, only eight were over-regulated: thy-1 cell surface antigen (*THY1*), Shisa Like-1, paired related homeobox 1 (*PRRX1*), alpha-2 subunit of collagen type IV (*COL4A2*), unc-5 netrin receptor B (*UNC5B*) and NAD(P)H quinone dehydrogenase 1 (*NQO1*). They constructed a three-gene MSC score that was based on the primary three genes linked to progression-free survival (PFS), *COL4A1,* natriuretic peptide receptor 3 (*NPR3*) and integrin beta like 1 (*ITGBL1*) [81]. *COL4A1* is an integral component of the basement membrane and is known to mediate the MGUS progression to MM [82]. Instead, *NPR3* and *ITGBL1* both appear to play important roles within the extracellular matrix, in tumor growth and carcinogenesis [83]; both were downregulated in this study [81]. In addition to *COL4A1*, other extracellular matrix genes such as *FBLN2*, periostin (*POSTIN*) and secreted frizzled-related protein 4 (*sFRP4*) were also overexpressed [82].

As mentioned earlier, MSCs can also differentiate in the adipocyte line, and it would appear that in MM patients this mechanism is also disrupted. A study reported that cell sub-populations of adipocytes expressing insulin-like growth factor binding protein-2 (*IGFBP2*), a specific marker of adiposity, were decreased in the bone biopsies of MM patients compare to those in MGUS and SMM patients [84].

In addition, co-culture experiments of MSCs from HDs with MM cells were also performed, showing a reduction in *IGFBP2* levels in MSC-MMs compared with those that had been cultured alone. The effect of cell–cell contact was also evaluated, which resulted in a marked suppression of *IGFBP2* in MSCs, whereas *IGFBP2* expression was just partially inhibited without cell–cell contact. Priming MSCs with MM cells suppresses their ability to differentiate into adipocytes and diminishes the expression of *IGFBP2* [84].

**Figure 1:**
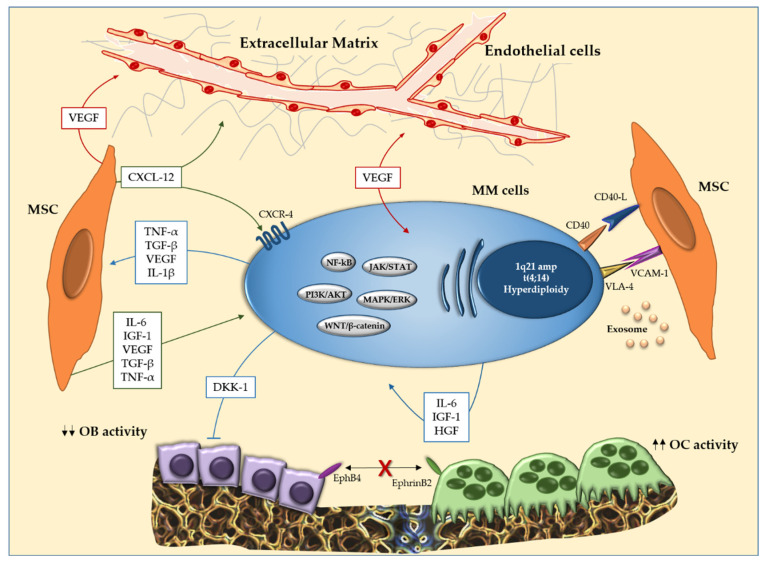
Interaction between multiple myeloma (MM) cells and bone marrow (BM) microenvironment cells. In the BM, the interaction between MM cells and mesenchymal stromal cells (MSCs) induces over-production of cytokines (IL-1β, IL-6 and TNF-α), soluble factors in the microenvironment and activation of molecular pathways (JAK/STAT, PI3K/AKT and WNT/β-catenin) that support survival and proliferation of MM cells. In addition, the cellular interaction results in activation of osteoclastogenesis and inhibition of osteogenic differentiation. EphrinB2 (expressed on OCLs) and EphB4 (expressed on OBs) also reduce osteoblastogenesis. Abbreviations: MM: multiple myeloma; BM: bone marrow; MSCs: mesenchymal stromal cells; IL-1β: interleukin-1β; IL-6: interleukin-6; TNF-α: tumor necrosis factor-α; JAK/STAT: Janus kinase signal transducer and activator of transcription pathway; PI3K/AKT: phosphatidylinositol 3-kinase/protein kinase B signaling pathway; WNT/β-catenin: Wingless/β-catenin signaling pathway; OCLs: osteoclasts; OBs: osteoblasts.

Another hallmark of MM is the homing of MM cells in the BM. It is critically regulated by the C-X-C axis motif chemokine ligand 12/C-X-C chemokine receptor type 4 (*CXCL-12/CXCR-4*). CXCL-12 is produced by MSC and is the CXCR-4 ligand which is expressed in PCs. *CXCL-12* is highly expressed by MSCs of MM patients in the BM sites [85]. In addition, *CXCL-12* upregulates *VLA-4*, which modifies cell adhesion of MM cells to MSCs and cytokine secretion by MSCs [86]. Moreover, blocking *CXCR-4* led to significant inhibition of cell migration and homing [85]. All of this evidence, considered together, supports the crucial role of the CXCL-12/CXCR-4 axis in homing and disease progression (Figure 1).

### 4.3. MicroRNAs and Mesenchymal Stromal Cells in MM

MicroRNAs (miRNAs) are short endogenous noncoding RNAs consisting of 19-22 nucleotides that regulate gene expression. They work at the post-transcriptional level. They act by linking to mRNA targets across their 3′ untranslated regions and recruiting the RNA-induced silencing complex, with subsequent repression or enhancement of DNA translation [87]. Indeed, miRNAs are classically categorized between oncogenic miRNAs and tumor-suppressive miRNAs [88]. The first class encloses miRNAs with higher expression in tumor cells and contributing to tumor evolution, while the latter class usually refers to miRNAs that normally suppress the expression of proto-oncogenes, whereas in cancers they are downregulated [89]. Many studies have stressed the prominence of miRNAs in physiopathology of neoplasms including MM.

In this regard, miR-485-5p has been proposed as a prospective candidate for the modulation of senescence in MSCs from MM patients. When there was MSCs cell interaction with MM cells and when cell–cell contact was prevented, a reduction of the levels of this miRNA was observed [90]. A decrease in miR-223 levels in MSCs also emerged as a consequence of the interaction of MM cells with MSCs. In tandem with reduced expression of miR-223, co-culture of MSCs with MM cells also induced Notch signaling, causing an increase in Vegf and IL-6. These events resulted in reduced osteogenesis [74,91]. In the same study by Berenstein et al. [90], the levels of miR-221 have been assessed. After its interaction with MSCs, it may be a negative regulator in osteogenic differentiation [92]. The osteogenic altered differentiation, resulting from abnormal expression of certain miRNAs, was also confirmed by bioinformatics tools [92].

The miR-135b is upregulated by MM cells [93]. Indeed, overexpression of this miRNA was observed in MSCs cells of HDs when co-cultured with MM cells. After removal of MM cells, miR-135b levels reverted to normal. In addition, a miR-135b inhibitor showed increased osteogenic activity in MSCs from MM patients. In the same study, the gene expression profile of MSCs from MM patients was also screened, displaying particularly high levels of miR-135b, resulting in impaired osteogenesis [93].

Another miRNA implicated in osteogenesis is miR-203a-3p, which regulates the Wnt/β-catenin pathway. In fact, a recent study showed that the use of an inhibitor targeting miR-203a-3p induced osteoblastic differentiation in MM patients, eventually triggering the canonical Wnt signaling pathway [94].

Finally, the role of a miRNA with a known oncosuppressive role, namely miR-34a-5p, was also investigated [95]. Its upregulation has been shown to suppress osteolytic lesion formation because it promotes the formation of functional SMADs, which are proteins that modulate the activity of Tgf-beta ligands [95].

It has also been reported that macrophages, located in the BM, being an integrated stromal cell component, can also protect MM cells from melphalan and from spontaneously induced apoptosis [96]. In MM, the macrophages can be recalled and activated by inflammatory factors such as Vegf [97]. Indeed, two miRNAs, miR-155 and miR-125b, have been proved to be able to promote the physiological macrophage activation [98] (Table 1).

## 5. Molecular Aspects of Osteoblasts in Multiple Myeloma

MM is characterized by an increased activity in OCLs and a diminished number of OBs. In fact, it has been observed that OBs apoptosis is significantly increased due to elevated cytokine levels and physical interaction between OBs and MM cells [99,100]. The interaction was replicated in vitro by co-culture systems of MM cells with OBs progenitors, evidencing inhibition of osteoblastic differentiation [72].

In the following section we will examine osteoblastic suppression in more detail.

### 5.1. Deregulated Wnt Signal Pathway

**Figure 2:**
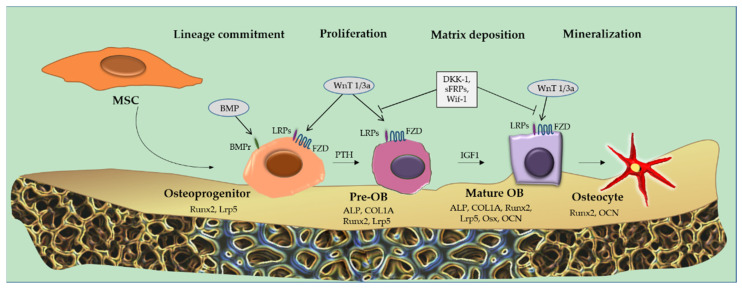
Osteoblastic suppression. Osteoblastic suppression occurs due to inhibition of the Wingless/int1 (Wnt) signaling/β-catenin pathway. The molecules responsible for the inhibition are dickkopf-related protein 1 (DKK-1), secreted frizzled-related protein (sFRP) and Wnt inhibitory factor (Wif-1), which bind to the low-density lipoprotein receptor-related protein receptors (LRPs). Runt-related transcription factor 2 (Runx-2) and Osterix (Osx), two fundamental genes for osteoblastic differentiation, are downregulated. Abbreviations: Wnt/β-catenin: wingless/int1 (Wnt) signaling/β-catenin pathway; DKK-1: dickkopf-related protein 1; sFRP: secreted frizzled-related protein; Wif-1: wnt inhibitory factor; LRPs: low-density lipoprotein receptor-related protein receptors; Runx-2: runt-related transcription factor 2; Osx: osterix.

A variety of molecules have been identified that negatively regulate the canonical Wnt signaling pathway by prompting phosphorylation and subsequent β-catenin degradation. Among these, the most highly recognized are Dkk-1, a member of the family of Dickkopfs proteins, the Wnt inhibitory factor (Wif-1), sclerostin and sFRPs. By binding to LRP5/6 receptors of the canonical Wnt pathway, these soluble factors are able to arrest OBs differentiation [101] (Figure 2).

MSCs from MM patients produce higher amounts of Dkk-1 [102], and MM cells were observed to show *DKK1* overexpression compared with MGUS patients and HD plasma cells [103].

The production of Dkk-1 by MM cells might be induced by the cell-to-cell adhesion of stromal cells and MM cells, which is also critical for Runx2/Cbfa1-mediated osteoblast inhibition [104]. A negative loop is triggered between MSCs, OBs and MM cells. Indeed, the undifferentiated MSCs produce IL-6, which is responsible for MM cell proliferation that secretes Dkk-1 [104].

The gene expression profiling further showed that high DKK-1 levels in the BM and peripheral sera of MM patients correlated with the development of focal bone lesions, [105,106].

Dkk-1 suppresses osteoblastogenesis and synergistically operates with sclerostin, resulting in OBs dysfunction [28]. Sclerostin is an inhibitor of the Wnt pathway, and it works by binding to LRP5/6. It is encoded by the *SOST* gene and produced by mature osteocytes that control bone formation and resorption [107]. In fact, higher levels of sclerostin were found in MM patients in association with abnormal bone remodeling [108]. An increase in this protein in osteocytes has also been demonstrated in mouse models [109,110].

As osteogenic differentiation of MSCs has also been proven to be initiated by the non-canonical Wnt pathway, some investigations have illustrated that MM cells are capable of blocking the non-canonical Wnt signaling pathway by inhibiting the expression of the Wnt5a receptor, Ror2, in human osteoblastic progenitors [33]. Indeed, in both MSCs and osteoblastic progenitors, *ROR2* mRNA levels were lower than in the same HD cells. Giuliani et al., in fact, reported that the overexpression of *ROR2* by lentiviral vectors boosted osteogenic differentiation of MSCs and mitigated the inhibitory effect of MM [33].

### 5.2. Runx-2

Osteoblastic suppression also happens because MM cells inhibit Runx2 activity in mesenchymal and osteoprogenitor stem cells [72]. Inhibition is induced by contact of MM cells and osteoprogenitor cells, exploiting the Vla4/Vcam-1 integrin system or other interactions such as Ncam–Ncam (CD56). One other soluble factor that inhibits osteoblastic differentiation is IL-7. Indeed, as shown by Giuliani et al., IL-7 produced by MM cells diminishes Runx2 activity on MSCs [72,111,112]. Furthermore, it has been proven that the up-regulation of IL-7 and Tnf-α drives the up-regulation of Gfi1, a transcriptional factor with repressor activity, which in turn reduces the activity of Runx2 in the MSCs and OBs of MM patients [73]. The involvement of IL-7 is also supported by the high IL-7 levels in the blood circulation of MM patients [113].

#### Epigenetic Regulation of *RUNX2*

A recent epigenetics-based study revealed heterochromatin silencing of the osteogenic factor promoter *RUNX2* [114]. As reported above, Gfi1 was over-expressed in patients with MM [115]. Other studies have been performed on MSCs from Gfi1 knockout or Gfi1 knockdown mice in murine precursors of OBs; these cells showed a better response to OB differentiation signals even if exposed to MM cells [73,116].

Moreover, Gfi1 suppression involves histone-modifying enzymes, namely histone deacetylase 1 (Hdac1), methyltransferases G9a and Ezh2 and lysine-specific histone demethylase 1 (Lsd1/Kdm1a) to target gene promoters [116,117]. Adamik et al. have highlighted enzyme recruitment by Gfi1, namely Ezh2, Hdac1 and Lsd1, to modify the bivalent signature of the *RUNX2* promoter to one mostly methylated at histone H3 lysine 4 trimethylation (H3K27me3). This could be determined after exposure to MM cells [114].

Interestingly, the use of some small molecules with an inhibitory role on Hdac1 or Ezh2 preserved the expression of Runx2, thereby promoting successful osteogenesis [114]. Another molecule employed in this framework was Xrk3f2, which, through the ZZ domain of p62 (Sequestosome 1), inhibits MM-induced upregulation of Gfi1, preventing the recruitment and binding of Hdac1 to the Runx2 promoter in pre-OBs [116].

Garcia-Gomez et al. found that the methylation status of the Homeobox family genes, involved in osteogenic differentiation, is different in MSCs of MM patients [118]. In fact, hypermethylated genes comprise positive regulators of OBs differentiation. In contrast, the hypomethylated genes are the negative regulators of osteoblastic differentiation. Among the hypermethylated genes, *RUNX2* or *NRP2* were noted, while among the hypomethylated genes, *sFRP2* or *NFATC2* were noted [118].

### 5.3. EphrinB2/EphB4

Osteoblastogenesis may also be suppressed by dysregulation of surface molecules that mediate the interaction between OBs and OCLs. It is well established that in MM, there is a marked disequilibrium toward OCLs compared with OBs. The molecules involved in such interactions are EphrinB2/EphB4. Eph receptors are tyrosine kinase receptors, enabled by the binding of specific ligands such as Ephrins. EphrinB2 is expressed in OCLs, while EphB4 in OBs and MSCs [119]. In MM patients, their expression is depressed. Low expression of both EphrinB2 and EphB4 lead to reduced osteogenic differentiation [120].

### 5.4. Role of Exosomes and Extracellular Vesicles in OBs Suppression and Translational Therapeutic Impact

Osteoblastic differentiation is also blocked by exosomes that are membrane-bound extracellular vesicles and produced in the endosomal compartment of most eukaryotic cells. Both in vivo and in vitro experiments, using the exosomes from the murine MM cell line 5TGM1, have blocked the differentiation of OBs [121]. These studies also showed the transfer of Dkk-1, which induced a decline in Runx2 and Osx in OBs [121]. In addition, exosomes from MM samples and MM cell lines carry Areg, the ligand of epidermal growth factor receptor (Egfr), which could be internalized by human MSCs with a consequent block of osteoblast differentiation [122].

Extracellular vesicles (EVs) are responsible for cell–cell communication, ensuring the information flow between PCs and MSCs or immune and endothelial cells [123,124,125].

Analysis of EVs from MM patients has highlighted their inhibitory role on osteoblastogenesis; in fact, it would appear that they reduce the expression levels of *RUNX2,* or that MM-derived EVs transfer Dkk-1 to OBs with subsequent reduction of Runx2 and Osx [121,122,126,127].

Several studies have corroborated the presence of miRNAs within MM-derived EVs [123,128]. An example is miR-129-5p, which is significantly higher in EVs of MM patients than in SMM patients. It blocks the activity of the transcription factor spi-1 proto-oncogene (*SPI1*) implicated in the differentiation of OBs; this blockade then determines the reduced activity of Alp, thus favoring the formation of OCLs [129].

Osteoblastogenesis can also be inhibited by miR-103a-3p, which appears to lead to severe bone disease, as confirmed in osteosarcoma, in which miR-103a-3p inhibits *AXIN2*, a component of the Wnt pathway [130]. The levels of miR-103a-3p in serum exosomes of MM patients were significantly elevated compared with SMM patients and HDs [131]. Zhang et al. also assessed PCs EVs levels in the peripheral blood of newly diagnosed MM patients, finding a positive correlation between EVs and osteolytic lesions. In the corresponding study, miR-103a-3p caused severe bone disease in vivo [127].

EVs also can carry non-coding RNA (ncRNA) [132], meaning that they are not translated into proteins. These ncRNAs can either have a cleaning function or check gene expression [133]. Indeed, there are several data that describe the roles of EVs and ncRNAs as influencers of MSCs differentiation during osteogenesis [122,127,129,134].

Numerous studies have examined the therapeutic potential of exosome secretion in MM to inhibit their contribution in cancer progression [135]. An example is the sphingomyelinase inhibitor, labeled GW4869 [136,137]. The use of this inhibitor in mice models showed an increase in cortical bone volume, reduced tumor burden and decreased markers of bone resorption. In particular, the use of GW4869 increased the activity of OBs and it sensitized the MM cells to proteasome inhibitor as bortezomib [121,138].

In addition, the exogenous administration of C6-cer ceramide dose-dependently enhanced the secretion of MM exosomes, but induced apoptosis and inhibited cell proliferation [139].

Moreover, exosomes and EVs not only can be involved in the OBs suppression, but also in drug resistance to principal therapeutic regimens in MM [140]. EVs derived from MM cells contained miR-146a and could be transferred into MSCs, causing the increased secretion of cytokines, including CXCL1, IL-6, IL-8, MCP-1 and CCL-5, and an enhanced MM cell viability and migration [141]. BMSC-derived EVs can also inhibit the effect of bortezomib, inhibiting MM cell apoptosis and the expression of Bcl-2 [142]. In addition, EVs derived from the BM of 5T33 mice contain *Mcp1, Mip1* and *Sdf1* that promote proliferation, survival and resistance to bortezomib in MM cell lines [142,143].

Finally, the inhibition of endocytosis of EVs from MM cells by MSCs prevents activation of pro-survival intracellular pathways, such as the *ERK/STAT* pathway, and restore drug sensitivity [144]. In fact, blunting the EVs endocytosis by chemical inhibitors (i.e., targeting heparin sulphate proteoglycans, actin, tyrosine kinase, dynamin-2, sodium/proton exchangers or phosphoinositide 3-kinases) sensitizes MM cells to bortezomib treatment in vitro and in vivo [145].

## 6. Conclusions

MM is characterized by the impairment of the BM niche influenced by clonal PCs.

It is known that MSCs and OBs from MM patients show several differences compared with HDs. In particular, these alterations were found in gene expression profiles, in the deregulation of some molecular pathways, in the cytokine release and in the action of miRNAs and exosomes. Indeed, during disease progression, there is an impairment of osteoblastic differentiation leading to the development of bone disease, a hallmark feature of patients with MM.

Understanding the molecular and functional characteristics of MSCs and OBs could contribute to developing new targeted treatments for MM patients.

## Figures and Tables

**Table 1:** The miRNAs related to the MSCs implicated in MM.

miRNA	Expression Pattern	Function in MSC	Target	Ref.
miR-34a-5p	Down	Osteolytic lesion formation suppression	SMADs	[95]
miR-125b and miR-155	Up	Promote physiological activation of macrophages, which results in drug resistance	IRF-4	[98]
miR-135b	Up	Compromised osteogenesis	SMAD5	[93]
miR-203a-3p	Up	Osteoblastogenesis suppression	Wnt/beta catenin pathway	[94]
miR-221	Up	Negative regulator in osteogenic differentiation	SMAD3	[90,92]
miR-223	Up	Impediment to osteogenic differentiation	VEGF and IL-6	[90]
miR-485-5p	Up	Modulator of the cell cycle and senescence status	―	[90]

## Data Availability

Not applicable.
